# Risk Management of Dairy Product Losses as a Tool to Improve the Environment and Food Rescue

**DOI:** 10.3390/foods8100481

**Published:** 2019-10-11

**Authors:** Beata Bilska, Danuta Kołożyn-Krajewska

**Affiliations:** Department of Food Gastronomy and Food Hygiene, Faculty of Human Nutrition and Consumer Sciences, Warsaw University of Life Sciences—SGGW, Nowoursynowska 159C St., 02-776 Warsaw, Poland; danuta_kolozyn_krajewska@sggw.pl

**Keywords:** food losses, risk management, food rescue nutrition, dairy products, bow tie analysis relationship diagram

## Abstract

“Food loss”, defined as food produced for human consumption, which for various reasons leaves the supply chain, can be assigned to a group of new risks. Irrational use of food constitutes a risk to the environment. Moreover, food losses represent a missed opportunity to improve global food security. The aim of this study was to develop a risk management model for dairy product losses using the example of ripening cheese. The necessary data to develop the model were derived from a survey that was conducted in five dairies located in Poland. Total losses for nine products amounted to 1.1% of the average annual production, which accounted for more than 5635 t per annum. The studies that were conducted allowed the identification of three management methods of food loss in dairies: reprocessing, hand over for feed, and disposal. The level of risk was defined as “high” with two suggested courses of action: prevention and tolerance. Risks must be prevented by eliminating any errors that may result in a product of inadequate quality. Another solution is to redistribute or sell products at a reduced price, which despite their reduced quality, are nevertheless suitable for consumption. To some extent, this risk must be tolerated.

## 1. Introduction

The term “food losses” is defined by Parfitt et al. [1] as a decrease in the amount of food at the production, post-harvest, or processing stages. In contrast, food losses at the retail and final consumption stages are called “food waste”.

The Food and Agriculture Organization of the United Nations (FAO) estimates that each year, approximately one-third of all food produced for human consumption in the world is lost or wasted. Global food security could be improved through the mitigation of food loss and food waste. Moreover, the limitation of food loss and food waste would be beneficial for the environment and natural resources. All people should have equal access to adequate safe and nutritious food that will meet their dietary needs [2,3]. Indeed, in 2017, nearly 821 million undernourished people lived across a number of countries [4]. Moreover, food insecurity (FI), defined as a socio-economic inability to obtain appropriate-quality food in adequate amounts [5], is also a problem in developed regions [6]. According to Jones [7], in Europe in 2014, 3.5% of people were considered severely food insecure (6.3% with moderate FI and 16.0 % with mild FI).

Risk, defined by Bernard et al. [8] as the probability of an event occurring and its impact on an entity, whether the impact is financial, physical, or on human health and wellbeing, is inscribed in the functioning of any organisation. Wrzosek et al. [9] draw attention to the bipolar nature of the concept of “risk” relating to food, which is interpreted both as a state of risk of food loss and as an opportunity to acquire it in advance, to turn it over to charity, and to prevent wastage.

Risk cannot be eliminated, but it can be identified and then managed, thus limiting poor decisions and losses. It should be noted, however, that over time, potential risks undergo radical changes. Therefore, organisations should use management models that take into account the growing diversity and complexity of the risks to the safety and quality of life of society. The probability of food loss can be classified as a group of “new risks” to which societies of the twenty-first century are exposed.

According to the Science and Technology Options Assessment, “food loss” means food produced for human consumption that leaves the supply chain for various reasons [10].

Although there is no clear and timely data on food losses in the supply chain, the amount is certainly significant. According to EUROSTAT estimates, in the EU-27 in 2006, nearly 35 million tons of food were lost at the stage of processing (with 39% accounted for in the loss structure households; 42% in food service and retail; and 19% in wholesale) [11]. In 2016, the EU Fusions project group indicated that the main sources of loss in the EU are households (53%) and processing (19%) [12]. However, in the EU-27, the largest amount of food is lost during the processing stage in Poland (over 6.5 million tons), the Netherlands, and Italy (6 million tons per year each) [13]. STOA estimates show that the share of processing in the total quantity of wasted food in Poland is 14% [10]. Food loss and waste mitigation is a critical means of addressing current and future economic, social, and environmental concerns to ensure enough food to feed the world’s growing population in a sustainable way [14].

Dairy plants produce at least several streams of liquid and solid waste. One of the main liquid waste products in the dairy industry is whey, which is considered to be hazardous to the environment due to the physicochemical properties, especially the high content of biodegradable organic substances, which make them hazardous when discharged into the environment. Their relatively easy biological degradation requires the consumption of large amounts of oxygen. Another liquid waste is buttermilk, which is a by-product of processing cream into butter and can be used for secondary processing as a semi-finished product for the production of food buttermilk or certain types of processed cheese [15]. At present, emphasis should be placed on the recovery of valuable nutrients from by-products that can be used for the production of food [2,16]. On the other hand, products that do not meet nutritional value requirements (e.g., non-standard fat content) or sensory characteristics (e.g., inappropriate salt content, spices) should be primarily directed toward consumption (they may be offered at a lower price or donated to charities) [16]. Considering that milk production contributes to the emission of significant amounts of greenhouse gases and the processes carried out in dairies are quite energy-intensive, all measures should be taken to direct products that are safe in terms of health toward human consumption. Only then should the next use be for animal feed and biogas production [17]. As noted by Chaboud and Daviron [18], when not consumed, food is redirected for animal feed and there is a reduction in the amount of other resources. Understanding the mechanisms of food loss will help identify appropriate measures to minimise it. The necessary tool could be ISO 31000:2018 Risk management—Guidelines [19], which is a set of principles, frameworks, and processes that are designed to meet the needs of every type of organisation, owing to its general and rational approach. According to the standard, each organisation should identify risks—the types, circumstances, or events that promote their occurrence and their potential consequences. After identifying and analysing risks, an organisation should identify those risks for which clear measures will be taken and those that will be accepted as residual risks [8].

The aim of this study was to develop a risk management model for dairy product losses using the example of ripening cheese. Our goals were: (1) to assess losses arising in the three stages, losses in relation to annual production, and the management methods; (2) to determine the significance and incidence of the causes of hazards; (3) to determine the causes and consequences of each of the three management methods. The research question was, what is the level of risk and which courses of action should be chosen to mitigate food losses?

This paper is organized into four sections. The research material and methods are described in Section 2. Section 3 reveals the study’s findings, which are discussed according to the body of literature. Finally, Section 4 contains the conclusions.

## 2. Materials and Methods 

### 2.1. Dairies

The survey was carried out between 2016 and 2018 in five dairies located in Poland. All plants belonged to one capital group, which is among the leaders in the dairy industry in Poland. Dairies differed in size and production range (Table 1) and were located in five voivodeships. In the selection of samples for research, a non-random sampling technique was used, namely targeted selection. In 2016, the capital group’s market share of dairy products sold was 18% in Poland. At all the sites, production took place using modern, fully automated production lines and manual work was performed on a small scale. The studied plants had certified HACCP systems and a Food Safety System Certification 22000 system equivalent to the British Retail Consortium and the International Food Standard.

### 2.2. Questionnaire

The research was carried out in each plant in two stages—consultation and detailed discussion of the questionnaire. In the second stage, the questionnaires were completed by Quality Representatives. The data that were collected concerned the production volume of each product; the amount of losses arising at the stages of pre-processing, processing, and customising; packaging and storage; and the management methods (Appendix A).

### 2.3. The Risk Management Model

The model is based on ISO 31000:2018 Risk management—Guidelines [19].

Detailed stages of the risk management process and the methods used are shown below:

#### 2.3.1. Risk Identification

The starting point was a detailed analysis of the available literature and the data collected in the dairies that were investigated. In the first stage, the following terms were defined:

- Hazard—product features reducing its quality (which consists of health safety, sensory characteristics, nutritional value, availability)
- Cause—the cause of hazard
- Risk of food loss—the probability of the occurrence of a hazard in the product, which may cause loss of food for consumption purposes or cause that food that is suitable for human consumption (but of lower quality than expected) to be subjected to reprocessing.

#### 2.3.2. Risk Analysis

Subsequently, the identified risks of food losses were analysed in order to understand them in detail. For this purpose, a relationship diagram was used to present the causes of hazards. To determine the significance and incidence of the causes of hazards, one of the questions that was included in questionnaire contained thirteen causes of hazards. A grading scale was used for assessment; the highest score awarded was 10, the lowest score was 1 (Appendix A). The next step was to develop a BowTie diagram, which presents the hazards and consequences. To determine the incidence of hazards, one of the questions that was included in questionnaire referred to this (Appendix A).

In monitoring the risk, a matrix of the probability of food loss (Table 2) and its consequences (Table 3) was developed. The starting point for developing the matrix of the probability and consequences was a detailed analysis of the available literature [19,20,21]. In order to determine the scale, the probability of the occurrence of losses from hazards in relation to the annual production was calculated. A five-point scale of the probability of the occurrence of hazard was adopted. A five-point scale for consequences assessment was adopted (Table 3). The consequences taken into account are related to product losses, financial losses, and the impact on the environment. Determination of the probability of the occurrence of an unfavourable event provides baseline information to determine the level of risk, which depends on the probability and consequences, and these parameters depend on the accuracy of the information input.

The values for the probability (P) and consequences (C) were transferred to the consequence/probability matrix. The values for the probability (P) and consequences (C) were calculated with the formula PC = (P) × (C). The filled-in consequence/probability matrix is presented in the “Results and Discussion” section of this article.

#### 2.3.3. Risk Evaluation

In order to evaluate risk, which has a significant impact on the decision-making process, the risk matrix was used to determine whether the expected risk was within the acceptance limits or whether it was outside these limits. The risk matrix defined the risk depending on the obtained value of PC. The risk levels were divided as: “low”, “medium”, “high”, and “very high”. For each of these risk levels, adequate decisions were indicated. If risk is not acceptable, further treatment is required.

#### 2.3.4. Risk Treatment

The results of the risk analysis formed the basis to decide what and to what extent the identified risks require that the organisation implement proper risk treatment algorithms and for their application to be prioritised. For this purpose, the risk treatment matrix was developed based on [19,20,21]. According to ISO 31000:2018 Risk management—Guidelines [19], options for treating risk may involve, for example, avoiding the risk, removing the risk source, changing the consequences, or retaining the risk by making an informed decision. The following risk treatment options were adopted: tolerance, prevention, and avoidance.

## 3. Results and Discussion

### 3.1. Risk Identification

Table 4 shows the volume of dairy production and the losses that occur in the three stages of the production process in the five plants that were studied. Total losses for nine products amounted to 1.1% of the average annual production, which accounted for more than 5635 t per annum. The percentage of food loss in the dairies that were studied was difficult to compare to the data that were included in the available literature because of differences in the size of samples. According to Mogensen et al. [22], food processing contributes to minimal food losses. Food losses in this area that could be used for consumption are estimated at the level of 1% to 2% of total production, while in Finland, Katajajuuri et al. [23] estimated the amount of food loss in dairies there at 33–43 million kg per year (an average of 3%).

The lowest average loss was reported at the stage of processing and customising and the largest was reported in the last link of the chain, namely packaging and storage. At this stage, the percentage of production loss was the most variable for particular products and ranged from 0.0002% to 11%.

In the next step, loss management was examined (Table 4). Products that are safe in terms of health, which do not meet the organoleptic (e.g., improper visual structure) or physio-chemical requirements (e.g., high water content), are most often used as raw materials for the manufacture of other products (e.g., cottage cheese, butter, and processed cheese). Residual milk powder, residues from packaging machines, conveyor belts, products with damaged packaging, and the like are used for feed. Products that cannot be used for animal feed due to contamination are sent to rendering plants. It should be noted that although a negligible percentage of losses are managed is this way in the plants that were studied, with this scale of production, it represents 19.1 t/year (Table 4). As Yasin et al. [24] noted, using unconsumed food for biogas plants is the most economical process. Another environmentally beneficial solution is animal feed [17]. However, food that is safe for consumption can be used to support people in need [25].

An important cause of food loss in dairies is the process of washing processing lines. The frequency of cleaning can be reduced by proper planning of the production sequence, where similar products are manufactured sequentially. However, this may result in the mixing of products, which cannot be sold in the ordinary way [16]. In the plants studied, so-called blended products are manufactured as a result of a change in the manufactured product range (e.g., changing yoghurt flavour from cherry to strawberry, adding chives to cottage cheese) without intermediate washing of the production line. Such products are packaged and used for consumption. This operation significantly reduces not only the waste of raw materials, but also resources, i.e., water and energy that would be used to wash the line. Over a year, the plants that were studied produced, on average, more than 14.5 t of blended yoghurt that was not intended for retail sale but was consumed by dairy workers or directed to people in need.

A particularly distinguished product group was ripened cheeses, for which the level of loss was 11% at the final stage of production as a result of, among other things, cutting and slicing (i.e., offcuts) as well as the production of cheese with inappropriate characteristics (e.g., visual structure, colour). For the above reasons, the risk management model for product losses was developed based on a product subject to the following management methods: disposal, hand over for feed, reprocessing into other goods.

### 3.2. Risk Analysis

#### 3.2.1. Relationship Diagram Illustrating the Causes of Hazards

**Figure 1:**
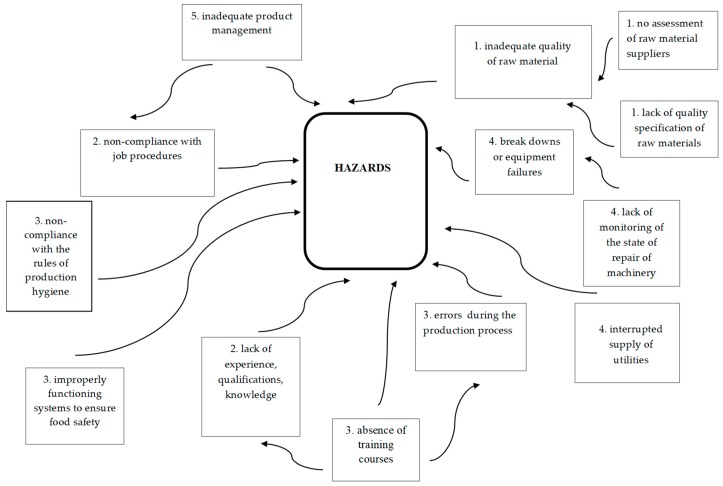
Relationship diagram illustrating the causes of hazards.

The relationship diagram below illustrates the causes of hazards (Figure 1). Five main causes were identified in the studied plants, which can be classified as follows: 1. materials, 2. people, 3. methods, 4. machines, and 5. management. This diagram not only defines the cause-effect relationships, but also the cause-cause relationships. For example, inadequate product management may be caused by insufficient qualifications, education, experience, and the lack of staff training. Inadequate product management may cause non-compliance with job procedures.

Table 5 shows the incidence of individual causes in the plants that were studied and their significance for the occurrence of hazards. On the basis of the data collected in the survey, it was found that all factors listed in the 10-point scale were highly significant (from 7 to 10 on a 10-point scale).

*1. Materials*

A fundamental role in food production is played by access to raw materials of appropriate quality. Each plant should have a quality specification for incoming raw materials, containing information relating to the required characteristics. The risk of food loss is related, for example, to cooperation with inappropriate suppliers. Each company should establish and maintain processes to identify, select, and evaluate its suppliers in order to continuously improve their ability to ensure that the products or raw materials they supply meet the needs of the organisation. Audits can be used to evaluate suppliers on the basis of the results. Many studies have shown different supplier assessment methods. Laeequddin et al. [26] developed a model to build confidence through risk assessment in the supply chain. Amid et al. [27] used fuzzy goal programming (FGP) to determine the appropriate number of orders to place with particular suppliers, taking into account the lowest prices and highest quality. Ghodsypour and O’Brien [28] used the Fuzzy Analytic Hierarchy Process (FAHP) for supplier selection, evaluating the following criteria: cost, quality, supplier profile, and risk factors. However, as noted by Trafiałek et al. [29], supplier evaluation methods are not well known in the Polish food industry, nor are they common in small and medium-sized enterprises in the European Union.

*2. People*

In any enterprise, the key factor that is responsible for the commission of errors is humans. As noted by Raak et al. [16], human errors in dairy companies can be connected with erroneous process control (mixed-up process parameters) or inaccurate handling of formulations (wrong starter culture, forgotten ingredients). The level of knowledge and qualifications among staff should be appropriate to the type of their activities. Lack of experience and relevant qualifications may result in errors leading to food losses. Therefore, it is important to improve the skills of workers through mandatory training, which should be carried out periodically in order to update and consolidate their knowledge [30]. However, as noted by Rossi et al. [31], employees in the food industry generally do not use their knowledge in practice.

*3. Methods*

The manufacture of adequate quality products is also determined by maintaining proper hygienic conditions during the entire manufacturing process. All companies operating in the food industry are obliged to comply with requirements concerning personal hygiene, washing and disinfection control, security, pest control, waste and sewage disposal, among others. The use of appropriate techniques, methods, and procedures in the production process ensures the safety of the food produced [32,33]. The result of the occurrence of irregularities can be a product that is dangerous to human health and consequently must be disposed of.

According to Hedberg et al. [34], the factors contributing to the spread of diseases by employees in the food industry include improper hygiene practices and levels of knowledge. Hand hygiene is one of the most important aspects in the prevention of disease transmission through food [35,36]. At the same time, research conducted by Green et al. [35] shows that food industry workers wash their hands too infrequently or incorrectly. While Pellegrino et al. [37] draw attention to how this can be caused by a lack of adequate motivation among the personnel, who despite knowing about hygiene, are nevertheless careless about it.

According to law that is currently in force, companies in the European food industry are required to implement mandatory systems to ensure food safety. Research has shown that errors in ensuring food safety are frequent [38]. Dzwolak [39] identified several factors causing difficulties in implementing the HACCP system in Polish small businesses. These factors include a lack of understanding of the guidelines, a lack of qualified and experienced personnel, and financial constraints. As observed by Bass et al. [40], the potential obstacles to the implementation of HACCP in Turkish companies include the lack of a prerequisite program, inadequate equipment, as well as inadequately trained and insufficiently motivated staff. According to Youn and Sneed [41], a lack of training is one of the biggest barriers to effective implementation of quality systems. Several studies point to the need to rebuild the system of training in the area of food safety, which should include workshops and training sessions [42,43,44]. Training can be effective in reducing the problems that are associated with food safety [44]. In addition, an integral part of human resources development should be an assessment of the training that is conducted. This is a way to assess the quality of training in terms of effectiveness and facilitates the achievement of the objectives pursued. According to Grover et al. [45], the main challenges in quality management in small food industry businesses include the cost of implementation, schedule of implementation, preparation of staff, a lack of quality culture, and no motivation among employees. 

To ensure the quality of food, it is necessary to select appropriate production methods and technical parameters and to ensure appropriate conditions during processing and storage. Most dairy products must be transported and stored at cool temperatures. Unexpected changes in temperature can lead to a deterioration in food safety and quality. [2]. The high share of these losses is related, among other factors, to insufficient training for workers in the cold chain and a lack of appropriate equipment [46]. Refrigeration or freezing equipment failure results in incorrect storage temperature and thus, endangers the safety of the raw materials and final products. In the studies conducted by Raak et al. [16], failures mainly concerned heating equipment. Such failures interfere with the control of the process and result in the production of non-sterile products or uncontrolled fermentation. Defects in sterile air generators can cause microbial contamination. A study conducted in 10 dairies by Richter and Bokelmann [47] showed that the main causes of loss are unforeseen circumstances, such as technical errors or product defects.

*4. Machines*

Each company must have adequate material resources, including equipment that is appropriate for the performed processes, to be able to operate efficiently. The technical condition of machines is extremely important and should be regularly monitored. Any equipment failures involve the loss of raw materials, semi-finished, or final products and can be prevented by regular inspections and maintenance, which should be part of any business management system. Moreover, a company should be protected against interruptions in the supply of power, e.g., by providing a backup source [48]. This conclusion is supported in the research conducted by Raak et al. [16] in 13 plants, including four in the dairy industry. Although power outages occur occasionally and are difficult to avoid, they may result in the loss of raw materials, for example, when the process requires an appropriate temperature (cooking, fermentation).

*5. Management*

The factors determining potential losses include improper product management, which results from the over-estimation of the volumes of production and orders, especially in peak sales, e.g., during holidays or long weekends. Although many manufacturing companies seek to avoid the accumulation of large stocks of products by supplying products “just in time”, surplus production cannot be fully eliminated. To counteract this phenomenon, monitoring and market research processes, demand estimations, processes of coordination, and cooperation between different sectors of the food chain [49] all must be implemented.

#### 3.2.2. Bow Tie Analysis for Reprocessing, Hand over for Feed, and Disposal

**Figure 2:**
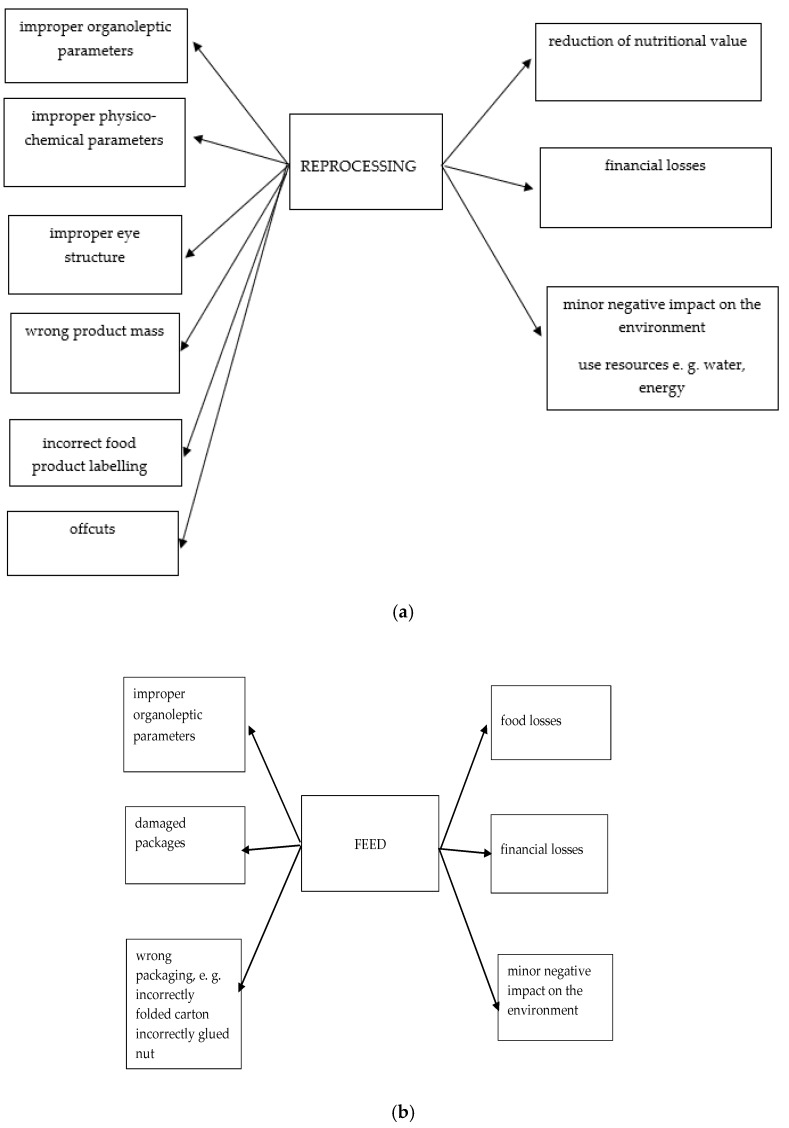

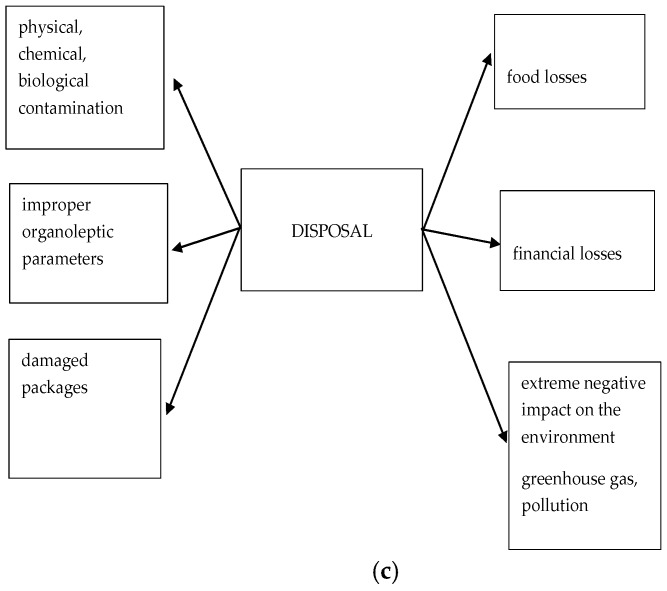
The bow tie analysis for reprocessing (**a**), hand over for feed (**b**), and disposal (**c**).

A bow tie analysis is a simple schematic way to describe and analyse the developmental path of an event from cause to consequence. It focuses on the barriers between the causes and the event as well as between the event and its consequences. This method serves to illustrate risk as well as the scope of possible causes and consequences of the given event [20]. Bow tie analysis was used to illustrate the hazards that may occur in the product and the possible consequences of risk (Figure 2a–c). Products that were safe in terms of human health were subjected to reprocessing (Figure 2a). The incidence of all hazards (except offcuts) resulting in reprocessing was 3 (for offcuts—10). These products did not meet requirements in terms of the senses, e.g., an incorrect appearance or physio-chemical properties. Currently, manufacturers sell products of the highest quality and those that are “imperfect”, for example, with aesthetic defects, are eliminated. Larger quantities may be redistributed as second choice products [16], however the transport of individual items is unprofitable.

Products that could not be reprocessed (e.g., due to contamination caused by damage to packaging) or products with incorrect organoleptic characteristics were used to produce feed (Figure 2b). The incidence of all hazards resulting in hand over for feed was 2. As Garrone et al. [50] noted, animal feed is often an appropriate alternative strategy; not only does it make it possible to avoid the cost of disposal, but it may produce revenue, although less than in the case of the sale of surplus food that was intended for human consumption. The positive impact of the hand over of surplus food to animal feed also has a positive impact on the environment due to saving resources (e.g., water, energy).

Products that are hazardous to human and animal health due to various errors in the process were disposed of (Figure 2c). The incidence of all hazards resulting in disposal was 2.

Although the causes of each of the three management methods are different, they all result in financial losses and impact the environment.

All three identified management methods of food losses are associated with a negative impact on the environment, although to varying degrees. Food losses have a negative impact on the environment in terms of the use of water, soil, greenhouse gases, pollution, and climate change [51]. As observed by Beretta et al. [52], the ecological importance of food loss not only depends on the quantity, but also the type of food, the stage in the food chain at which it is lost, and also on how it is recycled or disposed of. It is estimated that products that demand the highest consumption of natural resources and potentially have the greatest negative impact on the environment include beef and dairy products [53]. The food industry that uses the largest percentage of land is the production and processing of meat and milk (jointly), followed by the production and processing of cereals and eggs [3]. Reprocessing of products that are ready for consumption, but do not meet quality standards (e.g., improper visual structure), while likely to a lesser extent, nevertheless still burdens the environment due to the consumption of resources such as water and energy.

Financial losses result from the purchase of raw materials, which were subsequently reprocessed and, consequently, not sold at the expected profit. Economically, the most significant are financial losses incurred by businesses throughout the food chain, resulting from the lack of payment for the product that was manufactured. The cost estimated by FUSIONS for the EU 28 countries in 2012 was EUR 143 million, of which EUR 13 million accounted for processing [12]. Reprocessing involves additional costs (energy costs, staff wages, auxiliary raw materials, etc.) and as a result, the wholesale price of the product obtained is lower than the price of the product used. Cheese production is highly energy intensive. The average range of energy performance for cheese is 412 kWh/m^3^ raw milk, while the average range of energy performance for all dairies is 246 kWh/m^3^ raw milk [54]. Disposal not only involves additional costs, but also has the greatest negative impact on the environment. One of the indicators of the negative environmental impact of food losses is the total amount of uneaten food compared with the amount of water that is used to produce it. It is estimated that the amount of water wasted when food is discarded without being eaten amounts to about 250 km^3^ worldwide every year. Raw materials with the highest water demand throughout the entire food chain include cereals, fruit, meat, milk, and vegetables [3].

A lower nutritional value is due to the reprocessing of ripened cheese (melting), which was suitable for consumption (the vast majority of losses were formed at the final stage—Table 4). This process resulted in processed cheese that was characterised by reduced nutritional value compared with the raw material that was used (ripened cheese) (Table 6). The dairies in the study conducted by Raak et al. [16] declared a similar method of managing ripened cheese offcuts (residues generated during cutting, end pieces of cheese blocks), but drew attention to the fact that the demand for processed cheese is decreasing.

The values for the probability and consequences were transferred—for each of the three management methods—to the consequence/probability matrix (Table 7). The highest value is seen for reprocessing (15), while the remaining methods received 12 points each.

### 3.3. Risk Evaluation and Risk Treatment

The purpose of risk evaluation is to make the right decisions. One should compare the results of the risk analysis with the risk criteria to decide what action to take. Risk criteria are understood as benchmarks, relative to which the severity of the risk is determined. Risk treatment is useful to select and implement options that involve balancing the potential benefits derived in relation to the achievement of objectives against the costs [19]. If on the basis of numerical values the risk is considered high (reprocessing—15, feed—12, disposal—12), it is therefore unacceptable. Two risk treatment options were identified: prevention and tolerance (Table 8).

The first option, which at the same time is in line with “the waste hierarchy” [56], is “prevention”.

Based on the study, it was found that “loss prevention” involves the prevention of human errors by hiring suitably qualified and experienced staff, systematic training, implementation of quality systems, and the development of appropriate procedures. With proper production management, it is possible to avoid surpluses. Proper maintenance of machines reduces the likelihood of losses of raw materials or products. Products that are safe in terms of human health that do not meet quality standards should be donated to charities or sold at a reduced price instead of reprocessing. Unsold food should primarily be donated to non-profit organisations [1,17,25,57]. According to Muriana [58], donating to organisations can be an alternative that reduces the cost of management and storage and improves the company’s image. In research by Raak et al. [16], production facilities declared the donation of surplus food to charity as well as sale at factory outlets.

One cannot prevent all the negative situations; therefore, to some extent, risk must be “tolerated” in the following order:

- Reprocessing of food that is safe in terms of health but cannot be sold or donated for consumption (e.g., due to inadequate sensory attributes) and is eventually used in accordance with its intended purpose,
- Production of feed,
- Disposal: composting, energy recovery.

In summary, it can be stated that although the percentage of food loss in the investigated dairies amounted to only 1.1%, given the volume of production, the quantities are nevertheless significant. Therefore, it is worth considering the possibility of recovering food from this pool for consumption, taking into account that most of the losses were recorded in the last stage of production.

**Figure 3:**
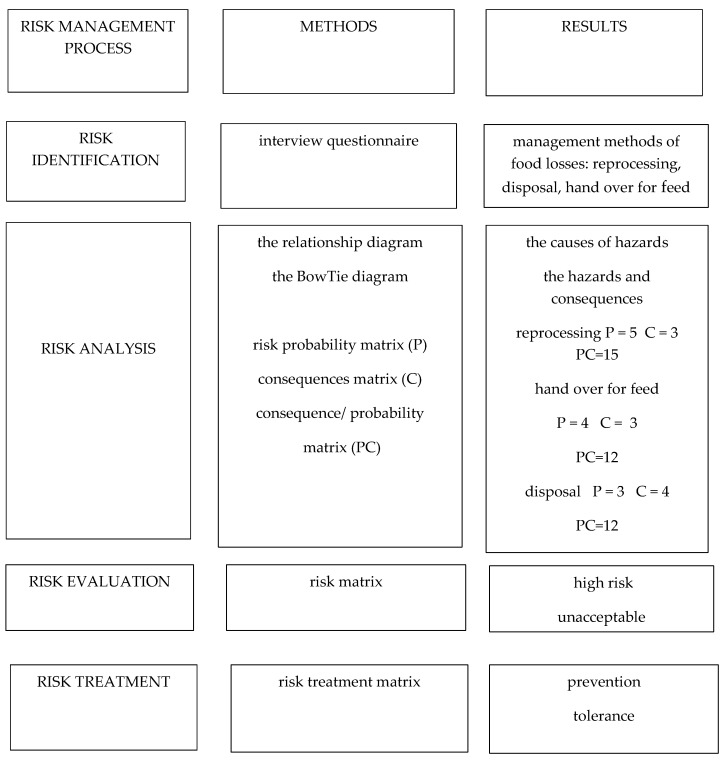
The risk management model for food loss: methods and results.

From a managerial perspective, our study yields the following implications:

- It draws attention to the scale of food losses and the need to take action for limitation;
- It can help in managerial decision-making about how to limit food losses and to use food in accordance with its intended purpose;
- It shows that donation for social purposes is beneficial from a waste management point of view and from an economic point of view;
- The proposed method can help the company’s manager to manage food losses;
- The risk management model for food loss that was proposed in this study is based on ISO 31000:2018 [19]. Figure 3 shows the use of risk management methods as well as the results that were obtained.

## 4. Conclusions

The studies that were conducted allowed the identification of three categories of management methods of food losses in dairies: reprocessing, turning over for feed, and disposal. The level of risk was defined as “high”, with two suggested courses of action: prevention and tolerance.

Risks must be prevented by eliminating any errors that may result in a product of inadequate quality. Another solution is to redistribute or sell at a reduced price products that despite their lower quality, are still suitable for consumption. This process of surplus food reallocation to those in need is called “food rescue nutrition” [59].

To some extent, the risk must be tolerated and products that are unsuitable for human consumption should be used to feed animals. Products that are hazardous to human health should be composted or sent to a biogas plant. The least favourable solution is the disposal of waste.

The risk management model for food loss that was proposed in this study is based on ISO 31000:2018 [19] and can be used in plants in different segments of the food industry.

We recommend that dairy companies use a risk management model in their operations so that the generation of food loss can be included in their operational processes. In our view, such actions can contribute to the reduction of food losses. The developed model is universal and can be used in other types of food processing plants.

A key limitation lies in describing one sector and collecting data from five dairies located in Poland. Further research should include various food production facilities. This descriptive study is helpful for providing information about food losses in dairies in Poland and is beneficial for both academicians and practitioners. From a methodological point of view, the proposed approach can be used in the risk management of food losses. However, additional research and data are necessary in order to develop the model for different segments of the food industry.

## Figures and Tables

**Table 1:** Characteristics of dairies in terms of annual volume of sales of milk, annual volume of production, and type of products.

Dairy	Annual Volume of Sales of Milk [t]	Annual Volume of Production [t]	Type of Products
Dairy A	557,000	366,000	All types of products listed
			below and cottage cheese
Dairy B	117,000	11,000	Cheese and curd cheese
Dairy C	153,000	16,000	Cheese and curd cheese, butter, and milk fats
Dairy D	317,000	75,000	Fermented products
Dairy E	225,000	37,900	Pasteurized milk, UHT milk, and ripened cheese

**Table 2:** Food loss risk probability matrix.

Assessment	Points	Description There are Reasonable Grounds to Believe That:
Rare	1	Food losses will not occur during the year or will be less than 0.001% of annual production
Unlikely	2	Food losses during the year will be more than 0.001% and less than 0.01% of annual production
Possible	3	Food losses during the year will be more than 0.01% and less than 0.1% of annual production
Likely	4	Food losses during the year will be more than 0.1% and less than 1% of annual production
Almost certain	5	Food losses during the year will be more than 1% of annual production

Source: Own study based on [19,20,21].

**Table 3:** Consequences matrix.

Assessment	Points	Description
Insignificant	1	- Results in insignificant product loss - Results in insignificant financial loss - Does not adversely affect the environment - Effects of the incident can be easily removed
Minor	2	- Results in minor product loss - Results in minor financial loss - Virtually does not adversely affect the environment - Effects of the incident can be removed
Moderate	3	- Results in moderate product loss - Results in moderate financial loss - Moderate negative impact on the environment - Effects of the incident are difficult to remove
Major	4	- Results in major product loss - Results in major financial loss - Negative impact on the environment - The effects of the incident are nearly impossible to remove
Extreme	5	- Loss of a large product quantity - Causes serious financial loss - Very negative impact on the environment - Effects of the incident cannot be removed

Source: Own study based on [19,20,21].

**Table 4:** The average annual volume of dairy production, the management methods, and the losses that occur in the three stages of the production process in the five plants studied [t] and [%].

Products	Average Annual Volume of Production	Losses Arising in the Three Stages						The Management Methods						Losses in Relation to Annual Production			
		Pre-Processing		Processing and Customising		Packaging and Storage		Reprocessing		Hand over for Feed		Disposal		Total	Re- Processing	Hand over for Feed	Dispo- sal
	[t]	[t]	[%]	[t]	[%]	[t]	[%]	[t]	[%]	[t]	[%]	[t]	[%]	[%]			
Pasteurized milk	174,667	17.5	0.01	1.8	0.001	1.8	0.001	0.0	0.0	21.1	100	0.0	0.0	0.012	0.0	0.012	0.0
UHT milk	195,000	19.5	0.01	1.9	0.001	1.9	0.001	0.0	0.0	23.3	100	0.0	0.0	0.012	0.0	0.012	0.0
Fermented products	14,667	1.5	0.01	0.2	0.001	0.2	0.001	0.0	0.0	1.9	99	0.02	1.0	0.012	0.0	0.01	0.002
Sour cream, cream	8667	0.9	0.01	0.09	0.001	0.09	0.001	1.06	98	0.02	2.0	0.0	0.0	0.012	0.01	0.002	0.0
Ripened cheese	49,600	5.0	0.01	0.50	0.001	5456	11.0	5352.2	98	90.2	1.65	19.1	0.35	11.01	10.8	0.18	0.03
Cheese and curd cheese	17,700	1.8	0.01	0.2	0.001	0.3	0.002	2.2	98	0.04	1.65	0.008	0.35	0.012	0.01	0.0002	0.002
Butter, milk fats	29,300	2.9	0.01	0.3	0.001	29.3	0.1	32.2	99	0.2	0.7	0.09	0.3	0.11	0.1	0.0008	0.0003
Milk powder	7032	0.0007	0.00014	0.01	0.0014	0.02	0.0003	0.04	100	0.0	0.0	0.0	0.0	0.0006	0.0006	0.0	0.0
Cottage cheese	9233	0.0	0.0	0.0	0.0	92.3	1.0	92.3	100	0.0	0.0	0.0	0.0	1.0	1.0	0.0	0.0
Total [t]	505,866	49.10		5.0		5581.9		5480		136.8		19.2					
Average [%]			0.008		0.0009		1.3		65.9		33.9		0.2	1.4	1.3	0.023	0.005

**Table 5:** The incidence of individual causes and their significance (average values in the five studied plants).

Causes	Incidence *	Significance **
1. Inadequate quality of raw material	1	10
1. No assessment of raw material suppliers	1	7
1. Lack of quality specification of raw materials	1	10
2. Lack of experience, qualifications, and knowledge	1	8
2. Non-compliance with job procedures	2	9
3. Non-compliance with the rules of production hygiene	1	10
3. Absence of training courses	1	10
3. Errors during the production process	1	10
3. Improperly functioning systems to ensure food safety	1	10
4. Break downs or equipment failures	2	8
4. Lack of monitoring of the state of repair of machinery	1	10
4. Interrupted supply of utilities	2	10
5. Inadequate product management (overproduction)	2	10

*—1—very rarely, e.g., once a year, 10—very often, e.g., once a week **—1—unimportant factor, 10—very important factor.

**Table 6:** Comparison of the nutritional value of rennet cheese and cream cheese.

Comparison of the Nutritional Value per 100 g of Products of Rennet Cheese and Cream Cheese	* Ripened Cheese	Cream Cheese *
Calories [kcal]	351.6	301
Protein [g]	25.7	13.5
Fat [g]	27.5	27
Calcium [mg]	734.9	367
Vitamin A [μg]	314.2	174
Vitamin E [mg]	0.5	0.5
Riboflavin [mg]	0.4	0.2

* average values for various types of cheese. Source: Own study based on [55].

**Table 7:** Consequence/probability matrix.

	Consequence	Insignificant	Minor	Moderate	Major	Extreme
Probability						
Rare						
Unlikely						
Possible					disposal 3 × 4	
Likely				feed 4 × 3		
Almost certain				reprocessing 5 × 3		

**Table 8:** Risk matrix and risk treatment.

Risk Matrix			Risk Treatment
Criteria		Evaluation	Risk Treatment Options
Level	Risk		
1–5	Low	Acceptable	1. Tolerance
6–9	Medium	Acceptable, requiring management decisions	1. Tolerance
			2. Prevention
10–16	High	Unacceptable	1. Prevention
			2. Tolerance
20–25	Very high	Unacceptable	1. Avoidance
			2. Prevention
			3. Tolerance

Source: Own study based on [19,20,21].

## Supplementary Materials

Supplementary File 1

## Appendix A

1. The Implemented Food Safety Management Systems

2. Characteristics of Production Lines

3. Please Complete the Table

Hazards	Incidence *	Management Methods
		
		
* 1—very rarely, e.g., once a year, 10—very often, e.g., once a week.		

4. Please Complete the Table

Products	Annual Volume of Production [t]	Losses Arising in the Three Stages [%]			The Management Method [%]		
		Pre-Processing	Processing and Customising	Packaging and Storage	Reprocessing	Hand over for Feed	Disposal
							
							
							
* 1—very rarely, e.g., once a year, 10—very often, e.g., once a week. ** 1—unimportant factor, 10—very important factor.							

5. Please Complete the Table

Causes	Incidence *	Significance **
Inadequate quality of raw material		
No assessment of raw material suppliers		
Lack of quality specification of raw materials		
Lack of experience, qualifications, and knowledge		
Non-compliance with job procedures		
Non-compliance with the rules of production hygiene		
Absence of training courses		
Errors during the production process		
Improperly functioning systems to ensure food safety		
Break downs or equipment failures		
Lack of monitoring of the state of repair of machinery		
Interrupted supply of utilities		
Inadequate product management (overproduction)		
* 1—very rarely, e.g., once a year, 10—very often, e.g., once a week. ** 1—unimportant factor, 10—very important factor.		

6. Are mixes produced in the factory? If YES, please provide what kind and in what quantities.
