# Supplementary material for: Risk Management of Dairy Product Losses as a Tool to Improve the Environment and Food Rescue
Source: Foods. 2019 Oct 11;8(10):481. doi: 10.3390/foods8100481 (PMC6835670; doi:10.3390/foods8100481)
Supplement: Supplementary File 1 [file foods-08-00481-s001.zip › Table, figure.v6/figure 1.docx]

1. no assessment of raw material suppliers

5. inadequate product management

1. inadequate quality of raw material

1. lack of quality specification of raw materials

4. break downs or equipment failures

2. non-compliance with job procedures

**HAZARDS**

4. lack of monitoring of the state of repair of machinery

3. non-compliance with the rules of production hygiene


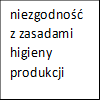


3. errors during the production process

4. interrupted supply of utilities

2. lack of experience, qualifications, knowledge

3. improperly functioning systems to ensure food safety

3. absence of training courses

Figure 1. Relationship diagram illustrating the causes of hazards
