# Supplementary material for: Risk Management of Dairy Product Losses as a Tool to Improve the Environment and Food Rescue
Source: Foods. 2019 Oct 11;8(10):481. doi: 10.3390/foods8100481 (PMC6835670; doi:10.3390/foods8100481)
Supplement: Supplementary File 1 [file foods-08-00481-s001.zip › Table, figure.v6/Figure 2 a,b,c,.docx]

improper organoleptic parameters

REPROCESSING

financial losses

reduction of nutritional value

improper physico-chemical parameters

wrong product mass

offcuts

improper eye structure

minor negative impact on the environment

use resources e. g. water, energy

incorrect food product labelling

**(a)**

improper organoleptic parameters

food losses

FEED

damaged packages

financial losses

wrong

packaging, e. g. incorrectly folded carton

incorrectly glued nut

minor negative impact on the environment

(b)

physical, chemical, biological contamination

food losses

DISPOSAL

financial losses

improper organoleptic parameters

extreme negative impact on the environment

greenhouse gas, pollution

damaged packages

**(c)**

**Figure 2**. The bow tie analysis for reprocessing (a), hand over for feed (b) and disposal (c)
