# Supplementary material for: Risk Management of Dairy Product Losses as a Tool to Improve the Environment and Food Rescue
Source: Foods. 2019 Oct 11;8(10):481. doi: 10.3390/foods8100481 (PMC6835670; doi:10.3390/foods8100481)
Supplement: Supplementary File 1 [file foods-08-00481-s001.zip › Table, figure.v6/Figure 3.docx]

RESULTS

RISK MANAGEMENT PROCESS

METHODS

RISK ANALYSIS

the relationship diagram

the BowTie diagram

risk probability matrix (P)

consequences matrix (C)

consequence/ probability

matrix (PC)

the causes of hazards

the hazards and consequences

reprocessing P = 5 C = 3 PC=15

hand over for feed

P = 4 C = 3

PC=12

disposal P = 3 C = 4

PC=12

interview questionnaire

RISK IDENTIFICATION

management methods of food losses: reprocessing, disposal, hand over for feed

high risk

unacceptable

prevention

tolerance

risk treatment matrix

RISK TREATMENT

risk matrix

RISK EVALUATION
