# Supplementary material for: Risk Management of Dairy Product Losses as a Tool to Improve the Environment and Food Rescue
Source: Foods. 2019 Oct 11;8(10):481. doi: 10.3390/foods8100481 (PMC6835670; doi:10.3390/foods8100481)
Supplement: Supplementary File 1 [file foods-08-00481-s001.zip › Table, figure.v6/Table.docx]

**Table 1. Characteristics of dairies in terms of annual volume of sales of milk, annual volume of production and type of products**

| Dairy | Annual volume of sales of milk  [t] | Annual volume of production  [t] | Type of products |
| --- | --- | --- | --- |
| Dairy A | 557 000 | 366 000 | All types of products listed in Table 4 |
| Dairy B | 117 000 | 11 000 | Cheese and curd cheese |
| Dairy C | 153 000 | 16 000 | Cheese and curd cheese, butter and milk fats |
| Dairy D | 317 000 | 75 000 | Fermented products, |
| Dairy E | 225 000 | 37 900 | Pasteurized milk, UHT milk, ripened cheese |

**Table 2.** Food loss risk probability matrix.

| **Assessment .** | **Points** | **Description**  **there are reasonable grounds to believe that:** |
| --- | --- | --- |
| rare | 1 | food losses will not occur during the year or will be less than 0.001% of annual production |
| unlikely | 2 | food losses during the year will be more than 0.001% and less than 0.01% of annual production |
| possible | 3 | food losses during the year will be more than 0.01% and less than 0.1% of annual production |
| likely | 4 | food losses during the year will be more than 0.1% and less than 1% of annual production |
| almost certain | 5 | food losses during the year will be more than 1% of annual production |

**Table 3.** Consequences matrix.

|  | | |
| --- | --- | --- |
| **Assessment** | **Points** | **Description** |
| insignificant | 1 | - results in insignificant product loss  - results in insignificant financial loss  - does not adversely affect the environment  - effects of the incident can be easily removed |
| minor | 2 | - results in minor product loss  - results in minor financial loss  - virtually does not adversely affect the environment  - effects of the incident can be removed |
| moderate | 3 | - results in moderate product loss  - results in moderate financial loss  - moderate negative impact on the environment  - effects of the incident are difficult to remove |
| major | 4 | - results in major product loss  - results in major financial loss  - negative impact on the environment  - the effects of the incident are nearly impossible to remove |
| extreme | 5 | - loss of a large product quantity  - causes serious financial loss  - very negative impact on the environment  - effects of the incident cannot be removed |

**Table 4. The average annual volume of dairy production, the management methods, and the losses that occur in the three stages of the production process in the five plants studied [t] and [%].**

| **Products** | **Average annual volume of production** | **Losses arising in the three stages** | | | | | | **The management methods** | | | | | | **Losses in relation to annual production** | | | |
| --- | --- | --- | --- | --- | --- | --- | --- | --- | --- | --- | --- | --- | --- | --- | --- | --- | --- |
|  |  | **pre-processing** | | **processing and customising** | | **packaging and storage** | | **reprocessing** | | **hand over for feed** | | **disposal** | | **total** | **re-**  **processing** | **hand over for feed** | **dispo-**  **sal** |
|  | [t] | [t] | [%] | [t] | [%] | [t] | [%] | [t] | [%] | [t] | [%] | [t] | [%] | [%] | | | |
| Pasteurized milk | 174,667 | 17.5 | 0.01 | 1.8 | 0.001 | 1.8 | 0.001 | 0.0 | 0.0 | 21.1 | 100 | 0.0 | 0.0 | 0.012 | 0.0 | 0.012 | 0.0 |
| UHT milk | 195,000 | 19.5 | 0.01 | 1.9 | 0.001 | 1.9 | 0.001 | 0.0 | 0.0 | 23.3 | 100 | 0.0 | 0.0 | 0.012 | 0.0 | 0.012 | 0.0 |
| Fermented products | 14,667 | 1.5 | 0.01 | 0.2 | 0.001 | 0.2 | 0.001 | 0.0 | 0.0 | 1.9 | 99 | 0.02 | 1.0 | 0.012 | 0.0 | 0.01 | 0.002 |
| Sour cream, cream | 8,667 | 0.9 | 0.01 | 0.09 | 0.001 | 0.09 | 0.001 | 1.06 | 98 | 0.02 | 2.0 | 0.0 | 0.0 | 0.012 | 0.01 | 0.002 | 0.0 |
| Ripened cheese | 49,600 | 5.0 | 0.01 | 0.50 | 0.001 | 5,456 | 11.0 | 5,352.2 | 98 | 90.2 | 1.65 | 19.1 | 0.35 | 11.01 | 10.8 | 0.18 | 0.03 |
| Cheese and curd cheese | 17,700 | 1.8 | 0.01 | 0.2 | 0.001 | 0.3 | 0.002 | 2.2 | 98 | 0.04 | 1.65 | 0.008 | 0.35 | 0.012 | 0.01 | 0.0002 | 0.002 |
| Butter, milk fats | 29,300 | 2.9 | 0.01 | 0.3 | 0.001 | 29.3 | 0.1 | 32.2 | 99 | 0.2 | 0.7 | 0.09 | 0.3 | 0.11 | 0.1 | 0.0008 | 0.0003 |
| Milk powder | 7,032 | 0.0007 | 0.00014 | 0.01 | 0.0014 | 0.02 | 0.0003 | 0.04 | 100 | 0.0 | 0.0 | 0.0 | 0.0 | 0,0006 | 0,0006 | 0.0 | 0.0 |
| Cottage cheese | 9,233 | 0.0 | 0.0 | 0.0 | 0.0 | 92.3 | 1.0 | 92.3 | 100 | 0.0 | 0.0 | 0.0 | 0.0 | 1.0 | 1.0 | 0.0 | 0.0 |
| Total [t] | 505,866 | 49.10 |  | 5.0 |  | 5,581.9 |  | 5,480 |  | 136.8 |  | 19.2 |  |  |  |  |  |
| Average [%] |  |  | 0.008 |  | 0.0009 |  | 1.3 |  | 65.9 |  | 33.9 |  | 0.2 | 1.4 | 1.3 | 0.023 | 0.005 |

**Table 5.** The incidence of individual causes and their significance (average values in the five studied plants)**.**

| **Causes** | **Incidence *** | **Significance **** |
| --- | --- | --- |
| 1. Inadequate quality of raw material | 1 | 10 |
| 1.No assessment of raw material suppliers | 1 | 7 |
| 1.Lack of quality specification of raw materials | 1 | 10 |
| 1. Lack of experience, qualifications, knowledge, | 1 | 8 |
| 1. Non-compliance with job procedures | 2 | 9 |
| 1. Non-compliance with the rules of production hygiene | 1 | 10 |
| 1. Absence of training courses | 1 | 10 |
| 3.Errors during the production process | 1 | 10 |
| 3.Improperly functioning systems to ensure food safety | 1 | 10 |
| 1. Break downs or equipment failures | 2 | 8 |
| 4. Lack of monitoring of the state of repair of machinery | 1 | 10 |
| 1. Interrupted supply of utilities | 2 | 10 |
| 1. Inadequate product management (overproduction) | 2 | 10 |

**Table 6.** Comparison of the nutritional value of rennet cheese and cream cheese.

| **Comparison of the nutritional value per 100 g of product of rennet cheese and cream cheese** | *** Ripened cheese** | **Cream cheese *** |
| --- | --- | --- |
| Calories [kcal] | 351.6 | 301 |
| Protein [g] | 25.7 | 13.5 |
| Fat [g] | 27.5 | 27 |
| Calcium [mg] | 734.9 | 367 |
| Vitamin A [μg] | 314.2 | 174 |
| Vitamin E [mg] | 0.5 | 0.5 |
| Riboflavin [mg] | 0.4 | 0.2 |

Source: Own study based on [55].

**Table 7**. Consequence/ probability matrix.

| **Consequence**  **Probability** | **Insignificant** | **Minor** | **Moderate** | **Major** | **Extreme** |
| --- | --- | --- | --- | --- | --- |
| rare |  |  |  |  |  |
| unlikely |  |  |  |  |  |
| possible |  |  |  | disposal 3x4 |  |
| likely |  |  | feed 4x3 |  |  |
| almost certain |  |  | reprocessing 5x3 |  |  |

**Table 8.** Risk matrix and risk treatment.

| **RISK MATRIX** | | | **RISK TREATMENT** |
| --- | --- | --- | --- |
| **Criteria** | | **Evaluation** | **Risk treatment options** |
| **Level** | Risk |  |  |
| 1-5 | low | Acceptable | 1. tolerance |
| 6-9 | medium | Acceptable, requiring management decisions | 1. tolerance  2. prevention |
| 10-16 | high | Unacceptable | 1. prevention  2. tolerance |
| 20-25 | very high | Unacceptable | 1. avoidance  2. prevention  3. tolerance |

Source: Own study based on [19-21].
